# Automated analysis of soft hydrogel microindentation: Impact of various indentation parameters on the measurement of Young’s modulus

**DOI:** 10.1371/journal.pone.0220281

**Published:** 2019-08-02

**Authors:** Steven Huth, Sandra Sindt, Christine Selhuber-Unkel

**Affiliations:** Institute of Materials Science, Biocompatible Nanomaterials, Kiel University, Kiel, Germany; Tufts University, UNITED STATES

## Abstract

Measurements of Young’s moduli are mostly evaluated using strong assumptions, such as sample homogeneity and isotropy. At the same time, descriptions of measurement parameters often lack detailed specifications. Many of these assumptions are, for soft hydrogels especially, not completely valid and the complexity of hydrogel microindentation demands more sophisticated experimental procedures in order to describe their elastic properties more accurately. We created an algorithm that automates indentation data analysis as a basis for the evaluation of large data sets with consideration of the influence of indentation depth on the measured Young’s modulus. The algorithm automatically determines the Young’s modulus in indentation regions where it becomes independent of the indentation depth and furthermore minimizes the error from fitting an elastic model to the data. This approach is independent of the chosen elastic fitting model and indentation device. With this, we are able to evaluate large amounts of indentation curves recorded on many different sample positions and can therefore apply statistical methods to overcome deviations due to sample inhomogeneities. To prove the applicability of our algorithm, we carried out a systematic analysis of how the indentation speed, indenter size and sample thickness affect the determination of Young’s modulus from atomic force microscope (AFM) indentation curves on polyacrylamide (PAAm) samples. We chose the Hertz model as the elastic fitting model for this proof of principle of our algorithm and found that all of these parameters influence the measured Young’s moduli to a certain extent. Hence, it is essential to clearly state the experimental parameters used in microindentation experiments to ensure reproducibility and comparability of data.

## Introduction

The ability of cells to sense and react to mechanical cues of their evironment has been largely accepted by the scientific community and is currently the focus of many research projects. For instance, stem cells differentiate in specific cell lines depending on the stiffness of their surroundings [1–5] and the efficiency of cardiomyocyte beating depends on substrate stiffness [6, 7].

These findings, however, pose new challenges for biomaterials research, as the elastic properties of these materials need to be controlled in order to fully predict and understand their interaction with cells. Such considerations are highly important for the development of implant materials as they require mechanical properties adaptable to their biological environments [8]. At the same time, *in vitro* experiments also need to consider mechanical aspects in order to properly mimic *in vivo* conditions. For decades, scientists have tried to create materials that mimic different extracellular environments [9, 10], as the objective to gain control over cellular behavior by manipulation of the cell’s surroundings would be of great interest for medicine, fundamental science, and tissue engineering [11, 12]. The influence of different biochemical cues, such as (very complex) protein structures or other chemical stimuli, have been extensively studied, but the influence of material mechanics on cell functions is not yet fully understood [13].

In order to characterize and manipulate a substrate’s elastic properties quantitatively, it is indispensable to possess a reliable and reproducable method to measure the material’s elasticity. However, not only substrate stiffness is essential, but in many cases it is also essential to quantify cell mechanics [14]. An example of this is that cancer cells can have a much lower stiffness than respective healthy cells [15], which is currently considered a highly significant fact in metastasis research. Nevertheless, state-of-the-art techniques, such as rheometer measurements or tensile tests, characterize the elastic properties of samples on a macroscopic scale [16], while cells interact with materials on a microscopic scale [17, 18]. It has already been discussed that microscopic indentation techniques like the atomic force micrsocope (AFM) often yield elasticity results with huge variations, and are more often discussed qualitatively instead of quantitatively [19].

During indentation experiments, a stiff indenter with known geometry is pressed into the sample while the force necessary to reach a certain indentation depth is measured. An elastic theory, which predicts the correlation of indentation depth and force, is used to calculate the Young’s modulus of the sample. The most commonly used elastic theory is the Hertz model [2, 3], which is valid for small indentation depths and reads as follows for a spherical indenter:

$$\begin{array}{c} F=\frac{4\sqrt{R}}{3}\;\frac{E}{1-\nu^{2}}\;d^{3/2} \end{array}$$ (1)

where *F* is the indentation force, *R* is the radius of the indenter, *E* the Young’s modulus, *ν* the Poisson’s ratio and *d* is the indentation depth [20].

Like most elastic theories, the Hertz model needs many strong assumptions such as substrate homogeneity and isotropy, or exact knowledge of the forces acting between the substrate and the measuring device [3, 21]. Especially for soft materials, which are commonly used for cellular experiments (the most prominent one is polyacrylamide (PAAm) [22]), many of these assumptions do not exactly hold [21]. PAAm is a microporous material with a high water content, consisting of a network of crosslinked monomer chains and is therefore neither homogeneous, nor isotropic, nor perfectly elastic [23–25].

Hence, it is very striking that the most commonly used elastic model to characterize elastic properties of hydrogels is still the Hertz model [2, 3, 26], even though many other models (such as the DMT or JKR [3, 27]) have been derived to overcome many of the Hertz models’ drawbacks. This already implies that the scientific community has to work with many approximations and that current theories do not perfectly match experimental situations. Although this makes it even more important to carefully state all the experimental parameters for elasticity measurements, published data often lack a detailed specification of these parameters. For example, when AFM is used for indentation experiments, the force up to which the material is indented or the speed of indentation, are often not stated, so that measurements from different research groups are not comparable. Indeed, studies that try to improve the Hertz model concentrate on the issue of describing more complex force interactions between the sample and the indenter (such as adhesive forces) [27] or on corrections for thin film substrates [26]. However, these improvements neither take into account the inhomogeneity nor the anisotropy of soft biocompatible materials.

The next step towards quantitative and comparable results does not only lie in a more sophisticated discrete theoretical description, but in more complex and statistically inspired experimental procedures. For this purpose, the AFM is the perfect tool, since it not only allows the implementation of indentation experiments down to piconewton precision, but it is also able to scan surfaces in the micrometer range, making it possible to measure the elasticity of substrates at many different positions. This overcomes some of the problems related to the inhomogeneity of surfaces such as topography differences or variations due to the indentation of a porous sample such as PAAm.

To accurately describe a sample’s elastic properties, it is necessary to record and evaluate a large number of force-distance curves. Once set up, the AFM can easily be used to automatically collect these indentation curves with a variety of different experimental parameters, such as indentation force and speed, at many different positions on the sample. The data analysis can also be automated, but so far, the influence of the indentation depth on the measured Young’s modulus is not included in standard AFM analysis software. However, the influence of the indentation depth on the elastic response of a material cannot be ignored. Cells, for instance, are known to probe their surroundings during decision making processes and the scientific community is already debating about how deep cells feel the mechanical properties of their substrates and how to adapt cell experiments to take this into consideration [19, 24, 28, 29]. Especially for hydrated materials, such as PAAm, this influence cannot be ignored when analyzing indentation curves, as the elastic properties of this hydrogel depend on indentation depth [29].

We here present a home written algorithm, that repeatedly fits an elastic model to a batch of indentation curves with a gradually increasing fit range. The curves were recorded with an AFM with spherical cantilever tips. The resulting Young’s moduli are plotted versus the indentation depth (we call this curve *E-d* curve) and the program checks where the Young’s moduli reach a plateau value. Within this plateau region, the Young’s modulus corresponding to the indentation depth, at which the respective AFM curve deviates the least from the fitted elastic model, is chosen as representative Young’s modulus. The algorithm evaluates many curves consecutively in order to automatically evaluate many different indentation data without ignoring the influence of the indentation depth on the Young’s modulus.

To prove the applicability of our new algorithm, we have conducted a systematic analysis of AFM indentation curves taken at many different positions on PAAm samples to measure the influence of setpoint force, indenter radius, sample thickness and indentation speed on the measured Young’s modulus. For simplicity, we chose the Hertz model for data evaluation but the algorithm can easily be adapted for the use of other, more sophisticated elastic models. When using the Hertz model, it would be faster to plot force versus indentation depth data in a double logarithmic plot. As the Hertz model then becomes a straight line with a slope of 1.5 (as log(*F*) = log(*αd*^3/2^) = 3/2 ⋅ log(*d*) + log(*α*), *α* as the parameter containing information on Young’s modulus), this would result in a simple linear fitting task. However, this approach does not work for elastic models containing sums instead of power laws (as log(*d*^3/2^ + *f*(*d*)), with f being some function depending on *d*, cannot be simplified). Many modern [26, 30] as well as classical theories (e.g. JKR or DMT [27, 31]) consist of sums. Thus, we decided to use the computationally more intensive approach of repeatedly fitting the Hertz model to the indentation data in a linear coordinate system in order to present a method that is compatible with other elastic theories than the Hertz model.

Our strategy to automate the analysis of indentation curves with respect to the influence of the indentation depth on the measured Young’s modulus allows a more comprehensive description of the elasticity of soft biomaterials. It will facilitate the computation of elasticity maps and consequently allow the measurement of the elastic properties of substrates on a spatial scale similar to that of cellular interactions. This method has a broad applicability as it is independent of the applied elastic theory and indenting device.

## Materials and methods

### Cantilever preparation

The AFM setups used in this study were a CellHesion 200 and a Nanowizard3 (JPK Instruments, Berlin, Germany) mounted on an IX73 or IX71 inverted microscope, respectively (Olympus Deutschland GmbH, Hamburg, Germany), which were equipped with a Progress MF cool camera (Jenoptik, Jena, Germany). The instruments were controlled with the CellHesion 200 and the Nanowizard Control Software (version 6.1.68A and version 4.3.5, respectively, JPK Instruments, Berlin, Germany). Tipless MikroMasch HQ:NSC35 and MikroMasch HQ:NSC36 (Innovative Solutions Bulgaria Ltd., Sofia, Bulgaria) cantilevers were utilized. Prior to use, each cantilever was calibrated for its spring constant and its sensitivity. The spring constants of the cantilevers were calibrated in air using the thermal noise method implemented in the JPK control software. The calibration for each cantilever was conducted five times at different positions on glass and the averaged spring constants were used in the analysis. In this study, spring constants were between 1.35 N/m and 1.89 N/m. Sensitivities were calibrated in water directly prior to each experiment.

A bead was glued to each cantilever with two component glue (UHU Plus Schnellfest, UHU GmbH & Co. KG, Bühl/Baden, Germany). Beads with diameters of 6.47 μm ± 0.32 μm (glass, microparticles GmbH, Berlin, Germany), 15.0 μm ± 1.0 μm (polystyrene, Merck Millipore, Burlington, USA; Scepter Test Bead Vial) and 21.82 μm ± 0.87 μm (glass, microparticles GmbH, Berlin, Germany) were used. The diameter values were manufacturer specifications of which we used the mean diameters for our calculations. Typical roughness values for such silica beads have been reported to range from 5 nm to 50 nm [32]. We assumed that the beads used in this study had similar roughness values as we always used the same bead type. The beads were washed once in ethanol and twice with double-distilled water. After each washing step, they were centrifuged for 10 min at 10,000 g and the supernatant was removed. Then the beads were deposited on a glass slide. To attach a bead, the cantilever was first dipped in glue and then pressed onto a single bead. After the glue was cured, the successful attachment of the bead was checked by imaging it from the side using a home-built cantilever holder in an IX71 microscope.

### Sample preparation

#### Pretreatment of glass slides

In order to promote PAAm gels to stick firmly to glass substrate holders, these holders need to be pretreated with methacrylate. 20 mm × 20 mm glass slides were cleaned three times with ethanol and double-distilled water. Then, they were incubated in sodium hydroxide (NaOH, 2.5 M) for 10 min and subsequently cleaned in an ultrasonic bath in double-distilled water for 10 min. The glass slides were rinsed with ethanol and incubated for 15 min in a mixture of 97% ethanol (absolute), 2% 3-(Trimethoxysilyl)propyl methacrylate (Sigma-Aldrich, St. Louis, USA) and 1% acetic acid (Sigma-Aldrich, St. Louis, USA). Afterwards, the glass slides were shaken in ethanol for 5 min at 80 rpm twice before baked at 120°C for 1 h.

#### Production of polyacrylamide

Polyacrylamide samples were prepared by mixing 1500 μl acrylamide (40%, BioRad Laboratories Inc., Hercules, USA), 900 μl bis-acrylamide (2%, BioRad Laboratories Inc., Hercules, USA), 25 μl ammonium persulfate (10 wt% in aqueous solution, BioRad Laboratories Inc., Hercules, USA) and 2575 μl double-distilled water. The mixture was degassed for 10-20 min in a desiccator. Afterwards, 7.50 μl N,N,N’,N’-Tetramethylethylenediamine (Sigma-Aldrich, St. Louis, USA) was added to the mixture to start the polymerization. 10 μl of the solution was deposited on a 20 mm × 60 mm coverslip and covered with a pretreated glass slide (we refer to these samples as “thin” PAAm samples). Bulk samples were prepared by adding the acrylamide solution to custom made Teflon molds with a depth of 1 mm or 2 mm and covering each sample with a pretreated glass slide. After 40 minutes of polymerization time, the coverslips were carefully removed from the thin samples and the bulk samples were removed from the Teflon molds. All samples were sticking thoroughly to the pretreated glass slides and were swollen in double-distilled water for at least four days prior to indentation experiments. The surface roughness of PAAm samples has been reported to be 40 nm [33].

### Indentation experiments

Prior to its use, each sample was fixed into a petri dish (TPP Techno Plastic Products AG, Trasadingen, Swiss) with biocompatible two component glue (Reprorubber, Islandia, USA). Furthermore, the sensitivity of each cantilever was measured on a hard reference material in double-distilled water prior to each measurement. For each indentation experiment, up to 10 force-distance curves were recorded at each of 25 different sample positions (with a distance larger than 15 μm between the positions), using the desired indentation speed and force setpoint. Sample rates of 2050 Hz and pulling lengths between 10 μm and 50 μm were employed for the indentation curves. PAAm samples were measured in double-distilled water.

### Data processing

AFM curves consist of an extend curve, where the cantilever is moved towards the sample until a setpoint force is reached, followed by a retraction of the cantilever (retract curve). An example of a curve is presented in the supporting information in S1b Fig. In this study, the extend segments of the measured indentation curves were used for the calculation of the Young’s modulus. First, the baseline and the tilt of the curves were corrected and subsequently, the tip-sample separation was calculated using the JPK data processing software (version 6.1.104, JPK Instruments, Berlin, Germany). A home written Matlab (Mathworks, version R2015a) algorithm was used to automatically calculate the Young’s modulus in the plateau region of each *E-d* curve (Young’s modulus as function of indentation depth). The software uses a least square fitting function to fit the Hertz model to the force data. The Young’s modulus and the contact point (position at which the indenter made contact with the sample) were used as fitting parameters. The Poisson’s ratio of PAAm was assumed to be 0.5 [30]. The fitting process was carried out repeatedly with increasing fit range, starting from the contact point to compute the Young’s modulus corresponding to different indentation depths. To reduce computation time, the total fit range was divided into 30 equally large parts. A standard deviation filter was used to check for groups of data points of the resulting *E-d* curve that present similar Young’s moduli. For this, the variance of all data subsets consisting of five consecutive data points (subsets overlapping by 50%) was calculated and variances below 5% of the maximum standard deviation were considered as data points with constant Young’s moduli. *E-d* curves that do not fulfill this criterion were discarded for further analysis. Only the first plateau after the contact point was considered. In this plateau, the Young’s modulus that corresponded to the fit range with the least chi squared (squared sum of residuals) was chosen as the representative value. At the end, the algorithm displays a table in which all relevant parameters are given (e.g. Young’s moduli, indentation depths, if plateaus are reached). Furthermore, the algorithm saves a table in which the number of saturated *E-d* curves is shown. From this table, the number of reliable values (i.e. the yield of a single measurement) can be calculated. Our algorithm was automatically applied to all indentation curves from one experiment. The algorithm is provided in the SI. If a huge variation of calculated Young’s moduli or striking values were observed in single experiments, the corresponding *E-d* curves and force-distance curves were controlled manually and values of Young’s moduli were discarded, if necessary. The number of remaining Young’s moduli was checked carefully as an insufficient number of values cannot represent the materials properties reliably.

All presented graphs were computed using Matlab R2015a or Origin 9.1.0 (OriginLab Corporation, Northampton, USA). For the boxplots, we used a convention in which each box represents 25% to 75% of the calculated Young’s moduli. The median is presented by a horizontal line. The crosses represent the minimum and the maximum of the distribution of Young’s moduli, whereas each square dot indicates the mean value of all measured Young’s moduli. Furthermore, the whiskers indicate outliers.

## Results and discussion

**Fig 1:**
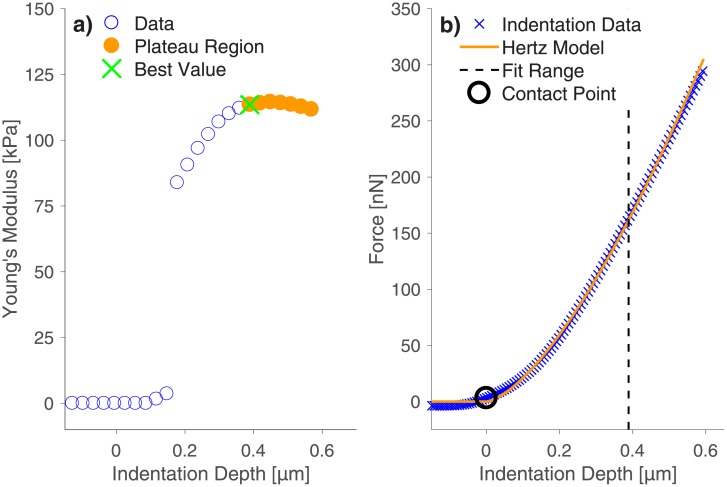
AFM indentation experiments on a thin PAAm sample. a) Calculated Young’s moduli as a function of indentation depth show that a high enough indentation depth is necessary to reach a Young’s modulus value that is independent of indentation depth. This is important for the reliability and comparability of elasticity measurements. In order to enable the analysis of huge amounts of data (many measurements on different positions), we created an algorithm that detects the plateau region of the *E-d* curves automatically. The orange dots represent the plateau region of the Young’s modulus determined by our algorithm, whereas the green cross shows the Young’s modulus with the lowest error of the Hertzian fit detected by our algorithm. Furthermore, the used fit range and contact point are displayed in the corresponding force-distance curve (b).

We indented a thin PAAm sample with a setpoint force of 300 nN, an indentation speed of 1 μm/s, and a 21.82 μm bead glued to an AFM cantilever with a spring constant of 1.35 N/m. We computed the Young’s modulus measured at different indentation depths by fitting the Hertz model with various fitting ranges to the indentation data. In Fig 1a we present an example of a resulting *E-d* curve. In such a typical *E-d* curve, the Young’s modulus increases up to an indentation depth of approximately 0.4 μm. Upon extending the fitting range to larger indentation depths, the Young’s modulus is almost constant. Values from this plateau region are assumed to describe the sample’s elastic behavior best. This means that it is important to indent a PAAm sample deeply enough to measure Young’s moduli independently of indentation depth. The depth necessary to reach a stable Young’s modulus might depend on several parameters such as sample roughness, indenter roughness or sample elasticity. Those dependencies are areas of further research and we highly recommend checking the *E-d* curves during indentation experiments until this subject is fully understood.

The indentation depths employed in this study are relevant for cellular research, as cells have not only been reported to deform substrates up to 0.5 μm in axial direction [34] and up to 2 μm in lateral direction [35, 36], but even feel rigid glass surfaces through 5 μm thick substrates [29]. Furthermore, these indentation depths are sufficiently small to avoid artifacts due to substrate compression or thin film effects, both of which are generally assumed to occur for indentations above 10% of sample thickness [21, 37, 38]. Our samples can be assumed to have a thickness above 25 μm, since we polymerized 10 μl of acrylamide solution between a 20 mm × 20 mm and a 20 mm × 60 mm glass slide (i.e. on an area of 400 mm^2^), resulting in a PAAm film of approximately 25 μm thickness. Additionally, the hydrogel samples are swollen in double-distilled water for several days prior to the indentation measurements, which results in a further increase of the sample volume (up to 2000% [10]) and thus a further increase in thickness.

Since an AFM cantilever can be treated as a spring with a known spring constant, higher indentation depths result from higher setpoint forces. Thus, a sufficiently high setpoint force has to be guaranteed during the elastic characterization of PAAm samples by indentation experiments. The observed dependence of Young’s moduli on indentation depth shows that the computation of such *E-d* curves is vital, as indentation curves alone do not display if the applied indentation force is sufficient to reach the plateau region of the Young’s modulus (see Fig 1b). This is a crucial aspect, because a sufficiently large indentation depth is the only way to guarantee repeatable and comparable results. The force-distance curve that belongs to the *E-d* curve of Fig 1a is presented in Fig 1b.

As a consequence from these findings, we applied an indentation force of 300 nN, as this was sufficient to reach the plateau region of Young’s moduli during the first experiments, but still computed each respective *E-d* curve with a homemade automatic Matlab algorithm for all further indentation experiments in order to check if enough force had been applied to reach the plateau in the *E-d* curve.

Since we had collected many different force-distance curves on different samples at many different sample positions (in order to avoid false results due to sample inhomogeneities), we created a Matlab algorithm to compute the *E-d* curve and to detect the plateau region of Young’s moduli automatically. This is achieved by using a local standard deviation filter, which checks the *E-d* curve for groups of consecutive data points with a variance below a user defined threshold. We chose this threshold to be 5% of the maximum standard deviation recorded for each curve. Only the first detected group of data points after the contact point is taken into consideration. From this part of the *E-d* curve, the software chooses the Young’s modulus that fits the force-distance curve best. The quality of the fit is quantified by the squared sum of the fitting residuals. In order to decrease computation time, we only computed the Young’s moduli for 30 different fit ranges between the contact point and the maximum indentation depth. In Fig 1a, the plateau region in the *E-d* curve is marked in orange and the best Young’s modulus, as determined by our algorithm, is highlighted with a green cross. Furthermore, the used fit range and the calculated contact point are displayed in the corresponding force-distance curve, which is presented in Fig 1b.

In S2 and S3 Figs of the supporting information, we prove that our approach yields the same results as the well-established approach of fitting only parts of the force-distance curve which present slopes of 1.5 when plotted in a double logarithmic coordinate system.

Classically, indentation experiments employ the retract curves, as plastic deformations alter the elastic response of a material during force loading [39]. However, upon retraction, the AFM cantilever sticks to soft materials such as PAAm, thus altering the retraction curve. This makes the determination of the contact point impossible. The knowledge of this contact point is very crucial, as it is needed to calculate the indentation depth from the cantilever position data provided by the AFM [40]. To prove that the use of the extend region is applicable, we first checked if the forces of 300 nN used for the abovementioned indentations deform the material plastically. We measured 100 indentation curves with a setpoint force of 300 nN at one position of the PAAm sample and analyzed if the resulting Young’s modulus changed during the course of the experiment. This was not the case, as is shown in S1 Fig a in the supporting information. Hence, plastic deformation of the PAAm samples upon indentation with 300 nN can be excluded and successive force-distance curves can be measured to describe the material more accurately. Furthermore, the extend and retract parts of the indentation curves do not differ in the regions relevant to the fitting process (an example is presented in the supporting information in S1b Fig). Finally, the absence of plastic deformation as well as the consistency of the extend and retract regions of the force-distance curves in the relevant fitting region justify our technically easier approach to evaluate extend curves.

### The influence of the indenter size

**Fig 2:**
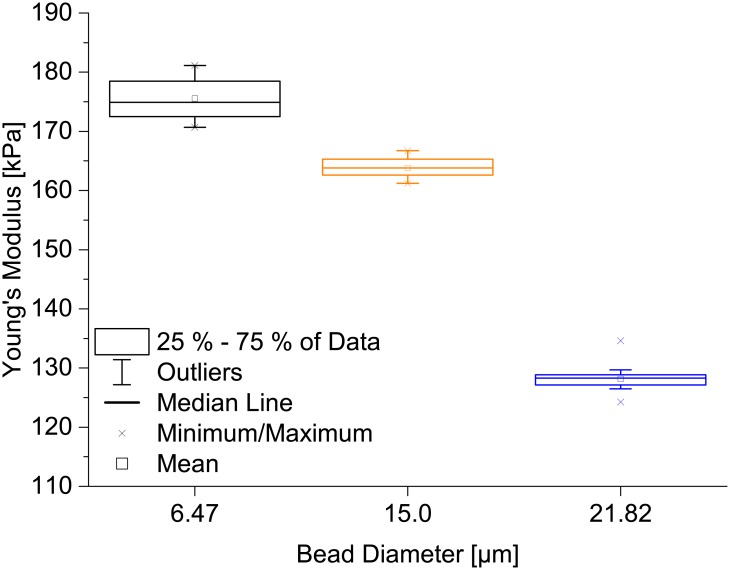
Effect of indenter size on the measured Young’s modulus for a PAAm sample. A thin PAAm sample was indented with beads of 6.47 μm, 15.0 μm or 21.82 μm diameter. Each box represents the distribution of Young’s moduli calculated from 30 indentation curves, taken at 3 different sample positions. A decrease of Young’s modulus is clearly visible when increasing the indenter diameter from 15.0 μm to 21.82 μm, which might be due to the fact that the indentation strain is larger for smaller indenters, resulting in the Hertz model to not be applicable anymore.

As a next step, we tested if different indenter radii result in different Young’s moduli. We indented a thin PAAm sample with beads having diameters of 6.47 μm, 15.0 μm or 21.82 μm glued to AFM cantilevers with spring constants ranging from 1.35 N/m to 1.89 N/m. As setpoint force we chose 300 nN except for the 15 μm bead, for which we employed 400 nN. Using an indentation speed of 20 μm/s, we collected ten force-distance curves for each of three positions on the PAAm sample for each bead size. The results are presented in Fig 2 and show a decrease of the measured Young’s modulus with increasing indenter diameter. The most probable explanation for this effect is that the Hertz model is only valid for small strains. The strain is expressed as *ϵ* = *a*/*R*, with *a* being the radius of the contact area between the surface and an indenter with radius *R* [41, 42]. Inserting $a=\sqrt{Rd}$, which is valid for the Hertz model [31] (*d* being the indentation depth), the strain is expressed as $\epsilon =\sqrt{d/R}$. Since a sample has to be indented to a certain depth, this condition sets a minimum for the applicable indenter radius. Hence, the bead with 21.82 μm diameter probably yields the most reliable results, as then the strain is smallest. This is additionally confirmed by the fact that the distribution of Young’s moduli for the 21.82 μm bead is the smallest. As a result of this experiment, we employed the 21.82 μm bead in all subsequent experiments. Another aspect necessary to consider before selecting a bead diameter is that horizontal sliding behavior of 10°-inclined cantilevers affects AFM curves stronger for lower length-to-tip-height ratios [43]. Thus, in order to minimize the influence of the indenter on the indentation measurement and since the bead size limits the resolution of the sample surface scanning, it is important to carefully choose a bead size that allows for reaching a plateau of Young’s moduli, but at the same time keeps the strain on the substrate small.

### The influence of sample thickness

**Fig 3:**
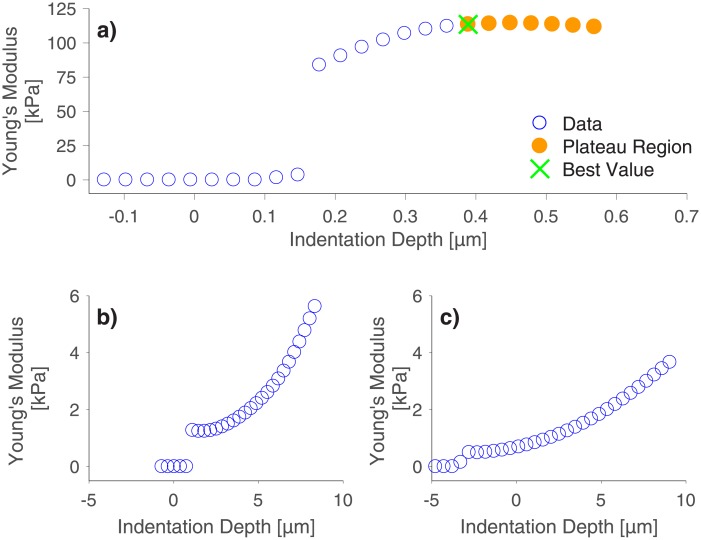
Influence of PAAm thickness on Young’s modulus. A thin (a) as well as a 1 mm (b) and a 2 mm (c) thick PAAm sample indented with a bead of 21.82 μm diameter. Although indentation depths up to 10 μm were reached for the bulk PAAm samples, no plateau region was detected. Only the thin sample shows a plateau value for the Young’s modulus for indentation depths above 0.4 μm. Generally, Young’s moduli decrease with increasing sample thickness.

We also investigated the influence of the PAAm sample thickness on Young’s modulus. To fabricate samples with different thickness, we produced two thick PAAm samples by polymerizing acrylamide solution in cylindrical molds of 1 mm or 2 mm depth and compared them with the previously shown thin PAAm sample. Exemplary *E-d* curves of the thin PAAm sample and of the thick PAAm samples are presented in Fig 3. The thin sample (a) shows a plateau in Young’s moduli upon an indentation of approximately 0.4 μm. The thick samples (b and c) on the other hand show no plateaus even for indentation depths larger than 10 μm and the *E-d* curves are shifted towards lower Young’s moduli ranges with increasing sample thickness. Such large indentation depths exceed the strain limit imposed by the Hertz model even for the largest indenter size employed in this study. Therefore, no Young’s modulus could be determined for the thick samples with our algorithm. This might be due to an increased shearing of the sample surface as cantilevers, which are normally installed into the AFM with an angle of 10°, do not move purely perpendicular to the indented surface, but also experience a force tangential to the surface. This can either lead to the cantilever sliding over the surface during the indentation or, if friction between the surface and the cantilever is high enough, the cantilever can shear the sample additionally to the indentation process [43, 44]. This effect might be enhanced for the thick samples as they were produced in Teflon molds, which can lead to a different surface structure compared to the thin PAAm sample. From these explanations, we conclude that the absence of a plateau region results from a combined effect of sample thickness and inclination angle of the cantilever. This hypothesis is supported by the fact that we did observe a plateau in the *E-d* curves when we indented the thick PAAm samples with a home-built indenting device where the horizontal movement was constrained. Exemplary graphs for these indentations of thick PAAm samples with a home-built indenting device are presented in the supporting information (S4 Fig). The AFM-intrinsic limitation could be overcome by employing special tilt-corrected cantilevers. Yet, all abovementioned observations underline the importance of observing how Young’s modulus changes with indentation depth.

### The influence of indentation speed

**Fig 4:**
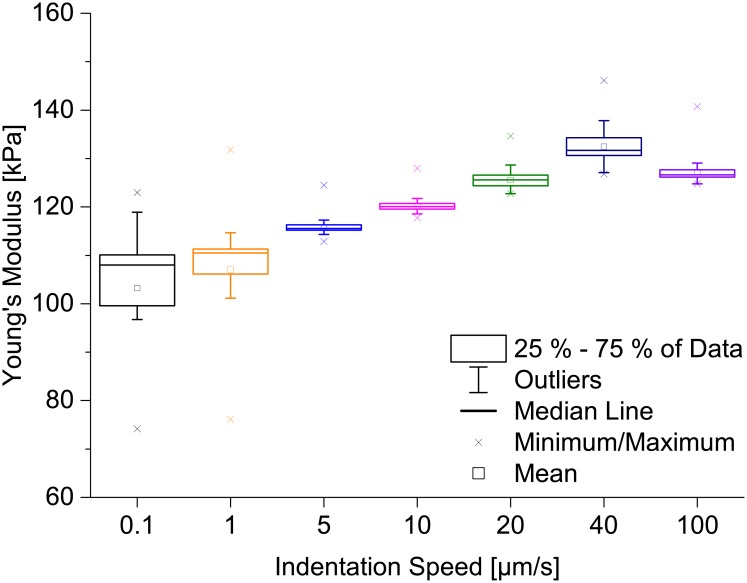
Dependence of Young’s moduli on indentation speed for a thin PAAm sample. For each of several indentation speeds ranging from 0.1 μm/s to 100 μm/s, 250 force-distance curves (10 curves at each of 25 positions) or 150 force-distance curves (10 curves at each of 15 positions) for 0.1 μm/s were measured and the distributions of Young’s moduli are presented as boxplots. The AFM measurements result in higher Young’s moduli for higher indentation speeds, except for 100 μm/s. The dependence of the Young’s modulus on the indentation speed is probably due to PAAm being viscoelastic [45]. Viscoelastic properties can be neglected on longer timescales [3] (i.e. for quasi-static indentation speeds such as 0.1 μm/s).

A further important parameter for hydrogel mechanics is the timescale of indentation. Therefore, we used our automated analysis to determine if the measured Young’s modulus is influenced by the indentation speed. We indented a thin PAAm sample with a bead of 21.82 μm diameter at 25 different positions on the sample using a variety of indentation speeds ranging from 0.1 μm/s to 100 μm/s, all using a setpoint force of 300 nN. For each position, 10 force-distance curves were recorded (i.e. 250 in total per speed or 150 for 0.1 μm/s). The Young’s moduli were determined from each of these curves using our algorithm. The results are shown Fig 4. The measured Young’s moduli increased with increasing indentation speed, except for measurements employing 100 μm/s, which resulted in slightly lower Young’s moduli than the data recorded with 40 μm/s. This was to be expected as it has been reported before that PAAm hydrogels are not purely elastic, but viscoelastic [45]. In principle, an indentation speed of 0.1 μm/s can be assumed as a quasi-static measurement for which viscoelastic behavior can be neglected. However, the number of force-distance curves that can be used for the calculation of Young’s moduli (i.e. the yield of usable curves) is an important factor, too, as discarding a huge number of force-distance curves by our algorithm means that the indentation data is not well described by the model. Consequently, a small number of analyzable curves cannot represent the sample accurately. Keeping these factors in mind, we suggest 1 μm/s as the optimum indentation speed for our specific system as the yield for a speed of 0.1 μm/s was the smallest in our experiments. Furthermore, the distribution of Young’s moduli for 1 μm/s is smaller, too. In conclusion, our results clearly show that the indentation speed is a parameter that cannot be ignored during the determination of elastic properties from indentation experiments.

### The influence of different sample positions and of consecutive curves at the same position

**Fig 5:**
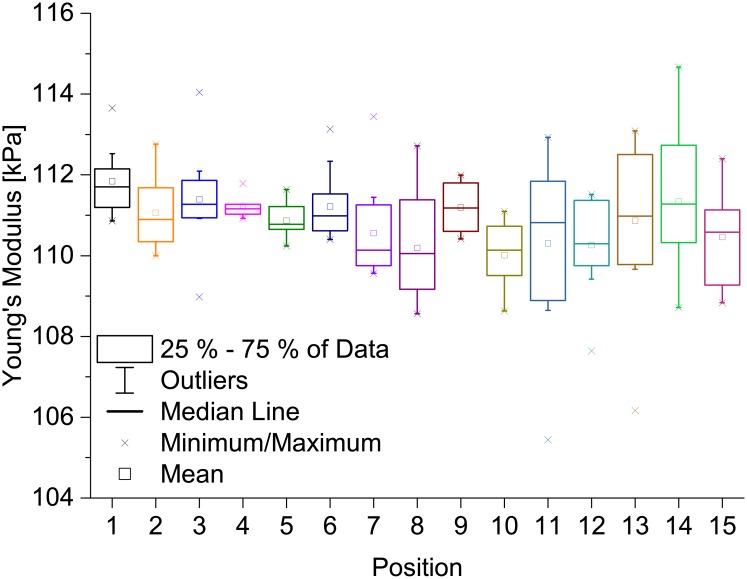
Young’s modulus on different positions on a thin PAAm sample. These measurements were carried out at an indentation speed of 1 μm/s using an indenter bead with a diameter of 21.82 μm and a setpoint force of 300 nN. The distributions of Young’s moduli are broad and mean values vary from one position to another. Measurements on different sample positions as well as several measurements at one position are necessary for reliably determining Young’s moduli.

Since we had collected many force-distance curves at many different positions on different samples, we decided to use the data to further compute the results for different positions on the samples. We compared the distributions of Young’s moduli measured on different positions with the distributions resulting from several indentations at single positions. In Fig 5, we present the distributions of Young’s moduli from 15 different positions on the PAAm sample measured with an indentation speed of 1 μm/s. It is very striking that the distributions of Young’s moduli measured on single positions are broad and that these distributions also vary from position to position. For instance, the distributions of Young’s moduli measured at positions 9 and 10 are clearly different, indicating that the deviation due to different sample positions cannot be ignored. On the other hand, the distribution at position 11 is very broad. The position dependence of Young’s modulus might be due to the fact that PAAm is a porous material with pore sizes up to the μm regime [25, 46], and that indenting the PAAm on its matrix can give different results than indenting it on pores. This means that it is necessary to evaluate many force-distance curves recorded at many different positions on the samples to receive reliable average values of the Young’s modulus, which once more emphasizes the necessity of sophisticated automatic evaluation algorithms such as the one presented here. Another aspect worth mentioning is that employing the AFM opens the possibility to evaluate the sample elasticity at different sample positions with submicron resolution, which is important to better describe how cells experience the mechanical properties of their surroundings, particularly if samples are mechanically inhomogeneous at the nano- and micrometer scale. Hence, describing a material with one single elasticity value is not sufficient for cell research and elasticity maps might be a solution for future comprehensive descriptions of the mechanical properties of cell environments. These, on the other hand, increase the need for automated indentation analysis even further, as finer resolutions will create larger amounts of data.

## Conclusion

We have developed and evaluated a new algorithm that automates the analysis of microindentation experiments by taking into account the dependence of Young’s modulus on indentation depth. We have demonstrated the applicability of this algorithm by a systematic analysis of the effect of several indentation parameters, such as indentation speed and indenter size, on the measured Young’s modulus of PAAm samples. For each experiment, many AFM force-distance curves were recorded at different sample positions. By applying the Hertz model, we have shown that indenter size, sample thickness and indentation speed all influence the elasticity results of indentation experiments on our soft samples. Consequently, these are relevant parameters that need to be stated and discussed in scientific publications to guarantee reproducibility and comparability of Young’s moduli measured in different research groups.

As a conclusion from our results, we propose a more sophisticated general experimental procedure to respect and exclude error sources in indentation experiments. First, the *E-d* curves of indentation experiments need to be computed to guarantee a high enough setpoint force to reach a plateau value of the Young’s modulus. This setpoint force should not be too high, though, as this would result in plastic deformation of the substrates. The indentation depth necessary to reach this region imposes a lower limit on the indenter size in order to keep the strain exerted to the sample low enough for the applicability of the Hertz model. Thus, the indenter size needs to be chosen carefully in order to maximize lateral resolution of elasticity maps while minimizing the sample strain. Tilt-corrected cantilevers are recommended in order to avoid sample shearing. Since the elastic response of PAAm samples during indentation varies for different indentation speeds, experiments must be carried out at well-chosen indentation speeds to suit the process of interest. To minimize viscoelastic effects as well as measurement errors, we propose to employ the smallest possible indentation speed in AFM based hydrogel indentation, while considering a sufficiently high number of analyzable curves (high yield). Several indentation curves have to be recorded at different sample positions to account for sample inhomogeneities and to minimize the variation of Young’s moduli on the sample. Since PAAm samples swell in water after fabrication, it is also very crucial to characterize samples only after having reached a stable state.

Our algorithm offers the possibility to automatically analyze several indentation curves while computing the respective *E-d* curve and choosing the Young’s modulus at an indentation depth that ensures stable and comparable results. The number of force-distance curves our algorithm can evaluate is only limited by computation power, thus opening up the possibility to also use it for recording elasticity maps. Our approach is independent of the applied elastic model and the indenting device, and the algorithm can easily be adapted to match the needs of various experimental situations.

## Supporting information

S1 Fig Indentations with 300 nN on one position of a thin PAAm sample justify the use of the extend curves. a) 100 indentations with a setpoint force of 300 nN and an indentation speed of 1 μm/s with a bead of 21.82 μm diameter on one specific position do not significantly alter the Young’s modulus measured on a thin PAAm sample over the course of the experiment. b) The extend and retract curve regions relevant to the fitting process are very similar for all indentation speeds (here shown for 1 μm/s) thus justifying the use of the extend curve to calculate Young’s moduli. We tested the extend part of the force-distance curve for linearity above 60 nN indentation force, which would indicate that the employed cantilever is too soft for sufficient sample indentation. Our data instead show that the curve does not show linear behavior (data not shown).
(EPS)

S2 Fig Determination of the fit range for the Hertz model by finding parts of the force-indentation curve with a slope of 1.5 in a double logarithmic plot. a) Exemplary force versus indentation depth data displayed in a double logarithmic plot. The indentation depth was calculated by subtraction of the contact point (determined from a complete Hertz fit of the entire force-distance curve) from the indentation data. The Hertz model is only applicable in the orange region of the force-distance curve. b) Here, the slope (Δ*log*(*force*)/Δ*log*(*indentation depth*)) of the curve from a) is plotted as a function of the indentation depth. A perfectly elastic material would result in a slope of 1.5, as then the Hertz model should perfectly fit. However, as PAAm is not perfectly elastic, we detected all data points with slopes between 1.25 and 1.75 (represented by the dashed orange lines). The corresponding region in the force-distance curve is marked in orange in a).
(EPS)

S3 Fig Comparison of Young’s moduli calculated with our approach to the results from the double logarithmic computation of force data. The distributions of Young’s moduli calculated with the log-log approach (presented in S2 Fig) as well as results employing our new method detecting plateaus in the *E-d* curves are represented by boxplots. We utilized a student’s t-test to verify that there is no significant difference between these distributions and with this, we proved that our approach yields the same Young’s moduli as methods of standard scientific practice.
(EPS)

S4 Fig Macroscopic indentation results in a plateau in the *E-d* curve recorded on a thick PAM sample. A 3 mm thick PAM sample was indented with a home-built indenter setup up to an indentation depth of 1 mm with an indentation speed of 0.5 mm/s and a spherical indenter with 6 mm diameter. Even though the high indentation depth necessary to reach a plateau in Young’s modulus allows only qualitative conclusions from this experiment, the calculated *E-d* curve verifies that a plateau region can be reached even for thick PAM samples. This means that using tilt-corrected AFM cantilevers could be a strategy to avoid artifacts from sample shearing (Fig 2). For details on the indenter, see supporting information of [10].
(EPS)

S1 Algorithm Please find here our Matlab algorithm that computes *E-d* curves for all indentation data in one folder and finds the Young’s modulus with least residual in the plateau region.
(M) Click here for additional data file.
